# DRIVERS OF SOCIAL WITHDRAWAL IN INDIVIDUALS WITH ALZHEIMER’S: AN ANALYSIS OF ONLINE PERSONAL NARRATIVES

**DOI:** 10.1093/geroni/igae098.2536

**Published:** 2024-12-31

**Authors:** Laura Hemmy, Yun Leng Wong, Mike Conway, Daniel Cabrera Lozoya, Kelvin Lim, Jude Mikal

**Affiliations:** University of Minnesota, Minneapolis, Minnesota, United States; University of Minnesota Twin Cities, Minnesota, United States; University of Melbourne, Melbourne, Victoria, Australia; University of Melbourne, Melbourne, Victoria, Australia; University of Minnesota, Minneapolis, Minnesota, United States; University of Minnesota, Minneapolis, Minnesota, United States

## Abstract

The relationship between social isolation and cognitive impairment is a pernicious cycle. On the one hand, social isolation is associated with an increase in risk of Alzheimer’s Disease (AD) and of AD progression; and on the other hand, the hallmark symptoms of increased cognitive and functional impairment have been shown to increase social withdrawal among individuals living with AD. As researchers, care providers, and clinicians design interventions to interrupt this cycle, it becomes necessary to understand the lived experiences and perspectives of persons living with AD, including the emotional, cognitive, and sensory drivers of isolation and withdrawal. The preponderance of research on social isolation and AD focuses on correlations and risk prediction models. Our study expands on this foundation through a focus on the catalysts, motivations, and consequences of increased social isolation among individuals with AD. Using the personal narratives of 8 individuals living with early onset AD maintained in publicly available blogs, we use a grounded theory approach to develop a conceptual model describing the emergence, predictors, and consequences of social withdrawal among individuals living with AD. Analysis revealed 5 key themes: (1) sensory symptoms of AD and impacts on social engagement, (2) cognitive symptoms of AD and impacts on engagement, (3) evolution of existing networks of support, (4) elective withdraw from social networks, and (5) consequences of withdrawal for individuals with AD and their care providers. Themes are then situated within contemporary research on AD and AD care, and include insights into key points of intervention.

